# Idiopathic osteoporosis, Ehlers–Danlos syndrome, postural orthostatic tachycardia syndrome, and mast cell activation disorder in a 27‐year‐old male patient: A unique case presentation

**DOI:** 10.1002/ccr3.5887

**Published:** 2022-05-20

**Authors:** Cameron Rattray

**Affiliations:** ^1^ St. George’s University School of Medicine St. George Grenada, West Indies

**Keywords:** Ehlers–Danlos syndrome, hypermobility, idiopathic, mast cells, osteoporosis, postural orthostatic tachycardia

## Abstract

A young male patient presents with widespread pain and varying chronic inflammatory symptoms for three years and idiopathic low bone density for more than ten years. Based on the patient’s clinical history, the patient has been diagnosed with an hypermobile Ehlers–Danlos syndrome, postural orthostatic tachycardia syndrome, and mast cell activation disorder trifecta with affiliated inflammation‐induced osteoporosis.

## Graphical abstract

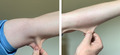

## 1 INTRODUCTION

The patient discussed in this report exhibited widespread algias and chronic inflammatory symptomology that went undiagnosed and persisted for more than three years and idiopathic osteoporosis and osteopenia that went undiagnosed for more than 10 years.

Osteoporosis is a metabolic bone disease characterized by a loss of bone mass and strength, resulting in increased fracture risk. The disease is rare and often idiopathic when discovered in young men.^1^

Osteoporosis, while often associated with decreased estrogen, vitamin D deficiency, and endocrine etiologies, can also be the result of collagen defects. Collagen is the protein supporting connective tissue, and hereditary collagen defects can result in multisystemic effects involving the cardiovascular, musculoskeletal, and integumental systems. Collagen may be defective in disorders such as hypermobile Ehlers–Danlos syndrome (hEDS) and is associated with low bone density. In certain patients, collagen defects are discovered through joint hypermobility tests. Joint hypermobility is a clinical sign of EDS published in the literature^2^ and may be evaluated with the Beighton scale, where a score of five (5) or more out of nine (9) defines hypermobility in post‐pubescent adults and before the age of 50 (A score of 6 out of 9 before puberty and 4 out of 9 after 50 years is required for a clinical diagnosis of hEDS according to the Beighton scale).^3^ Patients with hEDS often report daily pain, joint subluxations/dislocations, and skin and soft tissue complications.^4^

Following several years of clinical investigation, we report a rare case of osteoporosis and hEDS in a 27‐year‐old male patient with a constellation of symptoms affiliated with postural orthostatic tachycardia (POTS), mast cell activation disorder (MCAD), and hidradenitis suppurativa (HS).

### 1.1 Case presentation

The reported patient is a male, 27 years old, student, living in Texas, United States. The patient presented to an integrative wellness clinic with widespread pain and varying chronic inflammatory symptoms (including pyrexia of unknown origin, chronic mild lymphocytosis, pruritus, urticaria, constipation, and intestinal colic, and debilitating fatigue) that endured for three years. Additionally, the patient has exhibited idiopathic low bone density for more than ten years.

### 1.2 Patient history

The patient has a history of low bone density and was diagnosed with idiopathic juvenile osteoporosis at age fourteen following a complete fracture of his left hip at the intertrochanteric line and a subsequent positive bone densitometry (DEXA) scan.

In addition to the left hip fracture, and as a complication of the patient’s low bone density, the patient has a long orthopedic clinical history with fractures of the radius in both wrists, the medial epicondyle of the left elbow, the proximal tibia of the left knee, the talus of the left ankle, and several fingers and toes.

The patient’s most recent DEXA scan to date, in 2021, was reported as follows:

- Right femoral neck T‐score: −2.6
- Right Total Hip T‐score: −2.1
- Total lumbar spine T‐score: −2.1
- T‐score of −2.3 at L1
- T‐score of −2.1 at L2
- T‐score of −1.8 at L3
- T‐score of −2.0 at L4
- FRAX score was unavailable given the patient’s age≤40.

This DEXA scan found a 3.7% decrease in bone mineral density in the total lumbar spine since July 2019 and a 10.3% decrease in bone mineral density of the total right hip. Moreover, there has been no bone density improvement since his diagnosis at age fourteen.

Upon investigation into the patient’s bone loss, the patient’s serum parathyroid hormone, phosphate, magnesium, and calcium were all within normal limits and the patient’s serum vitamin D levels were low‐normal and improving. The patient’s other endocrine hormones including testosterone, thyroid, adrenocorticotropic hormone, and cortisol levels were all within normal limits. Additionally, the rates of degradation and production of bone products such as alkaline phosphatase, osteocalcin, CTX (serum cross‐linked C‐telopeptide of type I collagen), NTX (N‐terminal telopeptide), P1NP (total procollagen type 1 N‐terminal propeptide), and P1CP (procollagen type 1 carboxy‐terminal propeptide) were all normal. Lastly, a bone biopsy proved appropriate bone mineralization and trabecular arrangement.

The patient has not yet been treated with bisphosphonates or anabolic bone‐building medication due to the concerns about the long‐term consequences of these drugs provided the patient’s young age.

Provided the investigation into the patient’s peripheral osteoporosis, axial osteopenia and inflammatory symptoms, no overt etiology could be proved.

**FIGURE 1:**
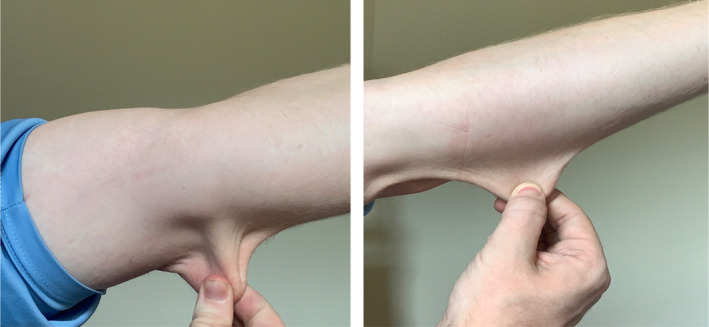
shows this patient’s skin hyper elasticity. Skin hyperelasticity is a clinical hallmark of hypermobile Ehlers–Danlos syndrome and falls under Criterion 2 Feature A, the systemic manifestations of a more generalized connective tissue disorder

In addition to the patient’s low bone density and associated orthopedic clinical history, the patient has a joint hypermobility history since childhood with previous orthopedic evaluation diagnosing the patient with ligament laxity following a traumatic left shoulder dislocation and subsequent subluxations. Patient evaluation of the musculoskeletal system proved joint crepitus, with slightly excessive finger and toe curvature. Additionally, with the expression of joint laxity, an evaluation of the patient’s face and skin evaluation was completed and proved mild skin elasticity and hypertrophic scar formation (Figure 1).

Upon further evaluation, the patient scored a seven (7) out of nine (9) after the Beighton criterion (Table 1) maneuvers were completed, consistent with joint hypermobility. The patient has met the standards for hEDS by qualifying for Criterion 1, Features A and C of Criterion 2, and met all of Criterion 3 (Table 2).

**TABLE 1:** Beighton Scoring System for assessing joint hypermobility^3^

Action	Right	Left
Passive dorsiflexion of fifth metacarpal bone >90°	1	1
Oppose the thumb to the volar aspect of ipsilateral forearm	1	1
Hyperextend to elbow >10°	1	1
Hyperextend the knee >10°	1	1
Put the hands flat on the floor without bending the knees	0.5	0.5
Total	9	

**TABLE 2:** Clinical diagnosis of hEDS requires the simultaneous presence of criteria 1 AND 2 AND 3^5^

**Criterion 1:** Generalized joint hypermobility (GJH) The Beighton score: ≥6 for prepubertal children and adolescents, ≥5 for pubertal men and women up to the age of 50, and ≥4 for those >50 years of age for hEDS. In individuals with acquired joint limitations (past surgery, wheelchair, amputations, etc.) affecting the Beighton score calculation, the assessment of GJH may include historical information using a five‐point questionnaire; if the Beighton score is 1 point below the age‐ and sex‐specific cutoff and the 5PQ is “positive” (= at least 2 positive items), then a diagnosis of GJH can be made [Notes and qualifications concerning this criterion in “The 2017 International Classification of the Ehlers‐Danlos Syndromes,” Malfait et al.]
**Criterion 2:** Two or more among the following features A, B, and C MUST be present (e.g., A and B; A and C; B and C; and A and B and C) **Feature A:** Systemic manifestations of a more generalized connective tissue disorder (a total of five must be present) 1. Unusually soft or velvety skin 2. Mild skin hyperextensibility 3. Unexplained striae such as striae distensae or rubrae at the back, groins, thighs, breasts, and/or abdomen in adolescents, men or prepubertal women without a history of significant gain or loss of body fat or weight 4. Bilateral piezogenic papules of the heel 5. Recurrent or multiple abdominal hernia(s) (e.g., umbilical, inguinal, and crural) 6. Atrophic scarring involving at least two sites and without the formation of truly papyraceous and/or hemosideric scars as seen in classical EDS 7. Pelvic floor, rectal, and/or uterine prolapse in children, men or nulliparous women without a history of morbid obesity or other known predisposing medical condition 8. Dental crowding and high or narrow palate 9. Arachnodactyly, as defined in one or more of the following: a. positive wrist sign (Steinberg sign) on both sides; b. positive thumb sign (Walker sign) on both sides 10. Arm span‐to‐height ≥1.05 11. Mitral valve prolapse (MVP) mild or greater based on strict echocardiographic criteria 12. Aortic root dilatation with Z‐score >+2 **Feature B:** Positive family history, with one or more first‐degree relatives (biological mother, father, brother, and sister) independently meeting the current diagnostic criteria for hEDS. **Feature C: Musculoskeletal complications (must have at least one)** 1. Musculoskeletal pain in two or more limbs, recurring daily for at least 3 months 2. Chronic, widespread pain for ≥3 months 3. Recurrent joint dislocations or frank joint instability, in the absence of trauma (a or b) a. Three or more atraumatic dislocations in the same joint or two or more atraumatic dislocations in two different joints occurring at different times b. Medical confirmation of joint instability at 2 or more sites not related to trauma
**Criterion 3:** all the following prerequisites MUST be met 1. Absence of unusual skin fragility, which should prompt consideration of other types of EDS 2. Exclusion of other heritable and acquired connective tissue disorders, including autoimmune rheumatologic conditions. In patients with an acquired connective tissue disorder (e.g., lupus and rheumatoid arthritis), additional diagnosis of hEDS requires meeting both Features A and B of Criterion 2. Feature C of Criterion 2 (chronic pain and/or instability) cannot be counted toward a diagnosis of hEDS in this situation. 3. Exclusion of alternative diagnoses that may also include joint hypermobility by means of hypotonia and/or connective tissue laxity. Alternative diagnoses and diagnostic categories include, but are not limited to, neuromuscular disorders (e.g., myopathic EDS, Bethlem myopathy), other hereditary disorders of connective tissue (e.g., other types of EDS, Loeys–Dietz syndrome, and Marfan syndrome), and skeletal dysplasias (e.g., OI). Exclusion of these considerations may be based upon history, physical examination, and/or molecular genetic testing, as indicated.

Additionally, the patient underwent an arduous investigative process to determine the etiology of his chronic pain, idiopathic osteoporosis, and other inflammatory symptoms, which were not explained by the hEDS diagnosis.

The patient reported follow‐up to various specialties, including endocrinology, immunology, rheumatology, allergy and infectious disease, hematology, oncology, gastroenterology, genetics, and cardiology. Following extensive testing including a whole‐exome screen and robust genetic testing, the patient has been diagnosed with gastroesophageal reflux, postural orthostatic tachycardia (following autonomic testing), mitral valve prolapse, mild aortic root dilation, tendonitis of the hip with bursitis, lumbar‐sacral early‐onset osteoarthritis without associated pain, idiopathic hypercalciuria, and hidradenitis suppurativa; however, there was no overt etiology for the patient’s chronic pain or osteoporosis discovered during the investigative process.

The patient’s whole‐exome screen, genetic testing, and laboratory workups allowed the patient’s healthcare team to rule out many differential diagnoses, including Marfan syndrome, Loeys–Dietz syndrome, TAAD, Lynch syndrome, multiple endocrine neoplasia types 1 and 2, hypertrophic cardiomyopathy, dilated cardiomyopathy, neurofibromatosis, hereditary paraganglioma‐pheochromocytoma syndrome, Tuberous sclerosis complex, Wilson Disease, Peutz‐Jeghers syndrome, Von Hippel Lindau syndrome, Juvenile polyposis, Long QT‐syndrome, familial adenomatous polyposis, osteogenesis imperfecta, Crohn’s disease, Rickets disease, osteomalacia, Paget’s disease, scurvy, Cushing’s disease, Cushing’s syndrome, Polycystic kidney disease, multiple myeloma, lymphoma, hyperparathyroidism (primary and secondary), hyperthyroidism, hypogonadism, and acromegaly, among others. Furthermore, fibromyalgia and chronic fatigue syndrome have been ruled out as potential etiologic causes to the patient’s symptomology.

Laboratory serum tests have proved the patient’s normal serum tryptase levels; however, he has exhibited a high level of serum immunoglobulin E in serum protein electrophoresis, which may be suggestive of secondary mast cell activation disorder. The patient has been considered for mast cell activation syndrome and mastocytosis given the nature of his inflammatory symptomology and his idiopathic low bone density; however, provided MCAD’s close relation to EDS and POTS^5^ and the presence of idiopathic inflammatory symptoms eased by dietary changes and histamine antagonists, in addition to the absence of any other known disorder that can account for the chronic inflammatory symptoms, secondary MCAD is a probable etiologic cause of the patient’s clinical presentation in the absence of elevated serum tryptase levels.

Seeking relief from the diverse clinical presentation the patient was experiencing, and upon recommendation by his primary care practitioner, the patient began a broad treatment regimen to improve his symptoms such that he could regain his desired quality of life.

Dietary changes were implemented and are now being followed closely. Consuming low histamine and low FODMAP foods proves helpful in ameliorating some of the patient’s inflammatory symptoms.

Moreover, while not a documented or proven therapeutic regimen, therapy was started with daily 6.0 mcg low‐dose naltrexone (LDN) due to its positive pain‐relieving effects in patients with chronic pain disorders and its role as a toll‐like receptor (TLR4) antagonist^6^ in addition to 20 mcg H1 antihistamines daily to relieve the suspected effects of MCAD. Furthermore, the patient began taking several vitamins daily, which include 5000 IU of vitamin D, and 500 mcg vitamin C, 1000 mcg folate, and 500 mcg glutathione.

The patient was instructed to maintain the aforementioned treatment regimen in addition to  low‐intensity physical activity, provided he use supportive braces and compression garments to help stabilize his joints. The patient has responded well to this therapeutic approach to symptom management; however, no improvement to his bone density has been ascertained.

Currently, the patient is followed up in the integrative clinic, with his endocrinologist and cardiologist, to monitor the patient’s bone density, POTS, and inflammatory conditions, including HS and the patient’s likely MCAD diagnosis. The patient was oriented to return every three (3) months to his integrative clinic for close monitoring and to undergo a once yearly DEXA scan to monitor his bone density.

Based on the patient’s extensive clinical history, exhaustive clinical investigation, and physical evaluation, the patient has been diagnosed with hEDS, POTS, MCAD, and HS. The patient has been asked to follow‐up with hereditary connective tissue disorder specialists and an immunologist with expertise in inflammatory bone diseases for further guidance concerning prognosis and therapeutics.

### 1.3 Social and family history

The patient is a non‐smoking graduate student with a robust and healthy diet.

The patient is a fraternal twin and in addition, and has a younger male sibling. The fraternal twin underwent surgical repair of his jaw at age 16 for temporomandibular joint injury repair, and this is not suspected to be connected to this report’s clinical presentation but due to traumatic injury. The patient’s younger male sibling displays very mild joint hypermobility but does not qualify for a hEDS diagnosis.

Neither parent has tested positive for a connective tissue disorder in whole‐exome sequencing. However, the patient’s mother does have normocalcemic hyperparathyroidism and post‐menopausal osteoporosis. Neither of which could be connected to this patient’s clinical presentation.

## 2 DISCUSSION

The presented case report is a complex presentation of systemic manifestations. The patient’s systemic clinical symptoms and extensive medical history align with hEDS as the primary etiology for the patient’s osteoporosis, in addition to much of his clinical history. hEDS, along with the other 12 EDS types, are closely affiliated with POTS and MCAD.^5^, ^7^, ^8^, ^9^, ^10^ While the three disorders are rarely found simultaneously in a patient, such a trifecta has been reported in the literature.^5^ The trifecta syndrome does account for the patient’s diverse clinical presentation.

To expand, the clinical characteristics of hEDS, while the least severe of EDS subtypes, can be severe.^11^ Chronic pain, distinct from acute joint subluxations and dislocations, is a serious and debilitating complication of the condition.^12^, ^13^ Easy bruising, skin hyperextensibility, functional bowel disorders, and cardiovascular autonomic dysfunction are common characteristics of hEDS. Aortic root dilation is present typically in a mild degree with no increased risk of aneurysm. Several common orthopedic injuries such as joint subluxations, joint dislocations, sprains, strains, iliotibial band syndrome, tendonitis, and bursitis are all common in hEDS, are likely secondary to joint instability, and may occur with minimal trauma in persons with hEDS. Additionally, hEDS is associated with early‐onset osteoarthritis as a result of chronic joint instability resulting in increased mechanical stress.^14^ Many of these complications are recognized within the reported patient’s clinical history.

Furthermore, there is limited and mixed evidence regarding low bone density in patients with hEDS. Bone density can be reduced up to 0.9 SD in individuals with hEDS compared to healthy controls, which may partly explain this patient’s decreased bone density. However, further evaluation of low bone density in individuals with hEDS should be explored.^15^, ^16^

It is critical to note the Ehlers–Danlos spectrum disorders are a family of multisystemic hereditary connective tissue disorders now comprised of 13 recognized subtypes, including classical, classical‐like, cardiac‐valvular, vascular, hypermobile, arthrochlasia, dermosparaxis, kyphoscoliotic, brittle cornea syndrome, spondylodysplastic, musculocontractural, myopathic, and periodontal, as designated by the most recent 2017 International classification system. Upon clinical investigation, hEDS (EDS Type 3) has less so been correlated to a specific genetic mutation.^15^ As it relates to this report, in a whole‐exome screen and subsequent genetic testing, the reported patient has tested negative for the classical and vascular EDS subtypes (Types 1 and 4, respectively) and does not meet the criterion or symptom presentation for the other EDS subtypes, outside of hEDS.

Keeping the negative results of an expansive clinical investigation in mind, another theoretical etiologic cause of the patient’s osteoporosis and osteopenia deserves further clinical investigation and exploration, particularly with the patient’s accompanying diagnosis of HS. For the purposes of this discussion, it is pertinent to note the role mast cell degranulation has in the proliferation and expansion of HS lesions and the chronic inflammatory nature of the disorder.^17^, ^18^ Mast cell degranulation occurs at a higher prevalence in other mast cell disorders, such as MCAD, and is affiliated with an increase in inflammatory markers.^19^

An inflammatory marker of particular interest in this report is interleukin 17 (IL‐17A), an inflammatory cytokine, which plays a primary role in chronic inflammatory skin conditions such as psoriatic arthritis and HS and is affiliated with mast cell degranulation.^17^, ^20^ Proinflammatory markers like IL‐17A play an important role in systemic and focal bone loss by inducing osteocytes and osteoblasts to secrete RANKL, which induces bone resorption.^21^

Given the patient’s clinical history of HS, we propound that IL‐17A may be circulating at higher levels within this patient, possibly due to mast cell degranulation. Without other explanations for the patient’s clinical presentation, it is reasonable to suggest a potential correlation between the patient’s skin condition and osteoporosis with that of MCAD, such that there may be an exacerbation of the patient’s bone loss.

However, without an overt etiology for the patient’s osteoporosis and osteopenia, hEDS and MCAD are the theoretical etiologies of the patient’s diminished bone mass; therefore, we cannot rule out an idiopathic nature of his low bone density.

It is also important to note the patient does display hypercalciuria, which may be associated with decreased bone mass.^22^ However, given the patient’s normal bone markers, endocrine hormones, and other serum laboratory tests, the patient’s hypercalciuria remains idiopathic in nature.

To expand briefly on the patient’s inflammatory symptomology, the patient’s fever of unknown origin, constipation, intestinal colic, and lymphocytosis may be the result of dysbiosis and leaky gut. Leaky gut and dysbiosis can be affiliated with EDS and MCAD, particularly in patients with dysautonomia.^23^, ^24^ The reported patient has exhibited some degree of dysautonomia with his POTS diagnosis; therefore, dysautonomia of the patient’s enteric system could explain these symptoms without an otherwise overt etiology. Patients with POTS and dysautonomia can be faced with more debilitating symptoms when associated with MCAD.^25^

Finally, the patient’s chronic pain is likely attributed to his hypermobility.^13^ This patient showed clinical signs of hEDS after specific Beighton criterion maneuvers and a review of his expansive clinical history. The patient’s additional symptoms of chronic fatigue and anxiety have confirmed the diagnosis of hEDS.

The process in diagnosing hEDS, POTS, MCAD, and HS is challenging due to the current diagnostic criterion, the complexity of patients’ profiles, the lack of definitive clinical signs, and the lack of overt laboratory or pathological findings. Additionally, the lack of consensus on treatment or treatment adaptation is also problematic in the long‐term care of persons with this disorder trifecta and associated low bone density.

The recognition of this specific phenotypic presentation of idiopathic osteoporosis with long‐term chronic pain and inflammatory symptoms could be a critical step in developing new therapy programs and discovering the pathogenesis of idiopathic osteoporosis in young men.

## 3 CONCLUSION

hEDS and MCAD may be associated with idiopathic osteoporosis in young men, and further research is needed to tie the disorders directly. hEDS, POTS, and MCAD together as a trifecta syndrome present as a complex and multisystemic disorder and may result in severe health conditions that may alter the patient’s quality of life. The major challenge in identifying this trifecta syndrome is an accurate diagnosis, as evidenced by this patient’s long‐term diagnostic process. A multidisciplinary approach should be encouraged and utilized regarding patient care, disease classification, clinical diagnosis, and the therapeutic approaches for disease management for persons with such a trifecta.

## AUTHOR CONTRIBUTION

CR wrote and corrected the article and provided pathologic photography and features.

## CONFLICT OF INTEREST

None. The authors do not have any reported conflicts of interest nor financial ties to report.

## ETHICAL APPROVAL

We confirm that the manuscript has been read and approved by all named authors. The protection of intellectual property associated with this manuscript had been in our consideration.

## CONSENT

Written informed consent was obtained from the patient to publish this report in accordance with the journal’s patient consent policy.

## Data Availability

All data relevant to the study are included in the article.
